# 796 Predictive Factors/Outcomes for Lower Extremity Amputations in Burn-Injured Adult Patients: 10 Year Review

**DOI:** 10.1093/jbcr/irac012.346

**Published:** 2022-03-23

**Authors:** Susan L Smith, Dominick M Curry, Andrew Rainey, Howard G Smith, Jacqueline Seoane

**Affiliations:** Orlando Health, Orlando, Florida; Orlando Health, Orlando, Florida; University of Florida, Gainesville, Florida; Orlando Health, Orlando, Florida; Orlando Health, Orlando, Florida

## Abstract

**Introduction:**

Lower extremity amputations (LEAs) in the burn population may occur urgently due to critical loss of tissue and perfusion or may proceed more slowly if viability remains uncertain. The existing literature describes amputations following electrical injuries; however, little is published discussing burn-injured patients who underwent delayed lower extremity amputations due to other burn mechanisms. Co-morbid illness and infections, patient/family resistance and provider delay in hopes of salvage may prolong time to amputation. Examining outcomes for burn patients’ suffering LEAs will delineate relationships among demographics, burn size, surgical timing, morbidity, mortality and length of stay (LOS) supporting the importance of early amputation.

**Methods:**

Single verified burn center conducted an IRB exempted retrospective medical records review of adult burn – injured patients, 18 years of age and older, admitted as inpatients from January 1, 2010 to December 31, 2020. Patients were identified using International Classification of Disease 9 & 10 and Current Procedural Terminology codes. Patients with non-burn related amputations or toe / partial foot amputations resulting from diabetes were excluded. The association between multiple variables and both day of first amputation and burn size was measured through linear regression, one sample t-test, and one-way analysis of variance (ANOVA).

**Results:**

The study population was 12 with 11 males, mean age 44, and one mortality. The majority (83%) suffered flame burns and 58% with mechanism of motor vehicle collision. Hospital day of first amputation ranged from 1-30, with a mean 11. Seven patients required vasopressors ranging from 30 minutes to 500 hours. For every 24 total pressor hours, day of first amputation increased by 1 day. Three patients (25%) had positive blood cultures and two (16%) had positive sputum cultures. Five patients (42%) had positive tissue cultures from the affected extremity. The diagnosis and treatment of infections were detailed and analyzed. Patients with positive tissue cultures were found to have waited an average 10.17 days longer for amputation than those without. Hospital LOS ranged from 31-126 days with mean of 54 days.

**Conclusions:**

Day of first amputation was the most impactful parameter, with longer time to first amputation associated with positive tissue cultures, mortality, vasopressor therapy and hospital length of stay. Limitations include retrospective nature, small sample size and reliance on electronic medical record and registry data. This study provided data to support earlier amputation for non-viable extremities, though additional research is clearly needed to better describe these relationships.

